# Phage-Displayed Peptides Selected to Bind Envelope Glycoprotein Show Antiviral Activity against Dengue Virus Serotype 2

**DOI:** 10.1155/2017/1827341

**Published:** 2017-09-10

**Authors:** Carolina de la Guardia, Mario Quijada, Ricardo Lleonart

**Affiliations:** ^1^Center of Cellular and Molecular Biology of Diseases, Instituto de Investigaciones Científicas y Servicios de Alta Tecnología (INDICASAT AIP), Building 219, Ciudad del Saber, Apartado 0843-01103, Panamá, Panama; ^2^Department of Biotechnology, Acharya Nagarjuna University, Guntur, India

## Abstract

Dengue virus is a growing public health threat that affects hundreds of million peoples every year and leave huge economic and social damage. The virus is transmitted by mosquitoes and the incidence of the disease is increasing, among other causes, due to the geographical expansion of the vector's range and the lack of effectiveness in public health interventions in most prevalent countries. So far, no highly effective vaccine or antiviral has been developed for this virus. Here we employed phage display technology to identify peptides able to block the DENV2. A random peptide library presented in M13 phages was screened with recombinant dengue envelope and its fragment domain III. After four rounds of panning, several binding peptides were identified, synthesized, and tested against the virus. Three peptides were able to block the infectivity of the virus while not being toxic to the target cells. Blind docking simulations were done to investigate the possible mode of binding, showing that all peptides appear to bind domain III of the protein and may be mostly stabilized by hydrophobic interactions. These results are relevant to the development of novel therapeutics against this important virus.

## 1. Introduction

Dengue virus is a growing public health problem worldwide as about 390 million people get infected annually and almost 96 million people develop clinical manifestations of the disease [1]. Other authors estimate that about 3.9 billion people from 128 countries share the risk of infection with this virus [2]. Dengue virus can produce a wide spectrum of clinical presentations, from asymptomatic or mild manifestation to a more severe life threating manifestation, known as dengue hemorrhagic fever (DHF) and dengue shock syndrome (DSS). Severe dengue can be deadly, especially in children, due to plasma leaking, respiratory distress, edema, severe bleeding, and organ impairment [3].

This virus is a* Flavivirus* that is transmitted by mosquitoes of the genus* Aedes*. The incidence of dengue infections has increased due to the spread of these mosquitoes into new regions [4]. DENV are present as 4 serotypes (DENV1, DENV2, DENV3, and DENV4) circulating in most endemic countries [5]. Dengue fever is considered the most rapidly growing mosquito-transmitted disease worldwide [6].

The dengue virion has an icosahedral symmetry, with diameter between 500 Å and 600 Å [7]. The viral genome consists of 11 Kbp single positive stranded RNA coding for a single polyprotein. The polyprotein is cleaved in the cytoplasm into several structural and nonstructural polypeptides [8]. The structural proteins include the capsid (C), premembrane (PrM)/membrane (M), and envelope glycoprotein (E), which are involved in the formation of the viral particle. The nonstructural proteins (NS1, NS2a, NS2b, NS3, NS4a, NS4b, and NS5) are responsible for the viral replication, assembly, and immune response escape [8].

E glycoprotein is the most important molecule during the viral entry process as it appears to be responsible for receptor recognition and attachment to the cell surface, triggering the clathrin mediated endocytosis and the subsequent fusion of viral and cellular membranes. This protein can interact with diverse cellular molecules; therefore, it is an ideal target for the development of new antivirals [9–12]. E glycoprotein is formed by three domains, plus a membrane proximal stem and a transmembrane anchor [13–15]. Domain I is formed by eight *β*-strand barrels containing two insertion loops. Domain II contains hydrophobic sequences that are conserved among all flaviviruses. These hydrophobic sequences, also known as fusion peptides, are responsible for the insertion of the rearranged E trimer into the cellular membrane during fusion [9, 14–16]. Domain III (DIII) is an immunoglobulin-like carboxyterminal domain, responsible for the initial cellular receptor binding [17]. Additionally, the DIII is the main target for neutralizing antibodies [18].

Due to the complexities of the immune response and the pathology generated by this virus, particularly during subsequent infections, the development of vaccines has been slow and there is only one vaccine that has been registered, which is still in the early stages of testing in several territories [19, 20].

Currently, there is no approved antiviral for the treatment of dengue infection. It is known that the duration of the viremia is short in dengue patients. However, since high viremia has been related to a severe onset of the disease, the use of antivirals at early stages of the disease may block the progression of the disease and accelerate the recovery of patients. Here, we proposed the use of dengue virus envelope (E) glycoprotein as a target to search for peptides that could interfere with the first step of the infection process. We selected the DENV-2 E glycoprotein and the DIII as targets for the screening random peptides displayed on M13 bacteriophages.

Phage display technology has been used in past years to identify peptides with specific binding activities to a variety of targets. This technique has been very useful to identify mimotopes, novel antivirals [21], peptidomimetic drugs [22], and many other applications.

Here we show that the DENV2 E glycoprotein, as well as domain III (DIII), is useful target for the identification of phage-displayed peptides with potential as novel antivirals. Here we describe three peptides that inhibit the viral infectivity and are not cytotoxic to the permissive cells. Fully blind docking simulations with these peptides suggest binding sites at domain III of the envelope protein, stabilized by predominant hydrophobic interactions. These findings open new possibilities to optimize and refine the design of new peptidic inhibitors of infection by this virus.

## 2. Materials and Methods

### 2.1. Cells, Viruses, and Peptides

Vero cells (CCL-81, ATCC, USA) were used for dengue 2 virus propagation, plaque formation assays, and cytotoxicity assays. These cells were grown in MEM with 10% FCS and 50 *μ*g/ml gentamicin, at 37°C and 5% CO_2_. Dengue virus, serotype 2, was a kind gift from Department of Research, Instituto Conmemorativo Gorgas de Estudios de la Salud (ICEGS), Panamá. Heptapeptide random library was obtained from New England Biolabs (USA). Synthetic peptides were obtained from Genscript (USA). Full DENV2 E glycoprotein (Cat. number Den-034) was obtained from ProSpec-Tany TechnoGene (Israel). Tissue culture, molecular biology grade, and general reagents were from Nunc and Sigma-Aldrich (USA).

### 2.2. Recombinant Protein Expression

Sequence coding for DENV2 E DIII (aa 289 to 405) was obtained from GenBank (complete genome NCBI Reference Sequence: NC_001474.2) and optimized for expression in* Escherichia coli* using an online tool (GeneOptimizer, Geneart). The optimized sequence was synthesized (IDT) and subcloned into NdeI-XhoI restriction sites of the expression plasmid pET-30b(+) (Novagen) using standard recombinant DNA techniques. This plasmid directs the inducible production of the DIII plus the amino acids Leu and Glu and a 6xHis tail at the C-terminal. This fragment of the E protein contains 125 amino acids and an approximate molecular weight of 14.2 kDa. The expression plasmid was verified by sequencing and transformed into* E. coli* strain BL21 (DE3) for expression. Single colonies were cultured in LB medium containing antibiotic at 37°C and 250 rpm and induced with 0.5 mM IPTG when OD_600_ reached 0.6. After 4 hours of induction cells were collected by centrifugation and kept at −20°C until purification. As expression of the recombinant protein was mostly at the insoluble fraction, further purification was done including inclusion body isolation, solubilization, and affinity purification in denaturing conditions, refolding, and dialysis.

### 2.3. Purification of Recombinant DIII

Frozen pellet from induced culture was suspended in cold lysis buffer (10 mM Tris-HCl pH 7.5, 5 mM benzamidine-HCl, 5 mM EDTA, 5 mM DTT, 0.3 mg/ml lysozyme, and 1 mM PMSF) and lysed by sonication on ice. The inclusion bodies were recovered by centrifugation, washed with 50 mM phosphate buffer, 5 mM EDTA, 200 mM NaCl, 0.5 M urea, and 1% Triton X-100, recovered as before, washed with 50 mM phosphate buffer, 1 mM EDTA, and 1 M NaCl, and solubilized in denaturing buffer (10 mM Tris-HCl pH 8.0, 100 mM NaH_2_PO_4_, 100 mM NaCl, and 6 M GuHCl). The solubilized inclusion bodies were purified using a Ni-NTA superflow cartridge (Qiagen). Following loading and washing with buffer C (6 M GuHCl, 100 mM NaH_2_PO_4_, and 100 mM HCl, pH 6.3), protein was eluted with buffer E (6 M GuHCl, 100 mM NaH_2_PO_4_, and 100 mM HCl, pH 4.5). Attempts to dialyze against PBS resulted in protein precipitation; therefore we tested several refolding conditions using a Protein Refolding kit (Pierce, USA). Finally, refolding was done by dilution into refolding buffer RB7 (1 mM GSH, 1 mM GSSH, 1 mM EDTA, 1.1 M GuHCl, 55 mM Tris-HCl, pH 8.0, 21 mM NaCl, and 0.88 mM KCl). The refolded protein was dialyzed against TBS, filter sterilized, and quantitated by BCA method.

### 2.4. In Vitro Virus Blocking Assay to Test Protein Refolding

Aliquots of refolded recombinant DIII, obtained from several refolding conditions, were first visually checked for aggregation. Those not showing aggregation were then tested in virus infection blocking experiments, to check for their ability to bind cellular receptors and inhibit these first steps in viral infection. Vero cells were plated in 96-well plates at 10^4^ cells per well in complete medium (MEM, 10% FCS, and 50 *μ*g/ml gentamicin). On the next day, cells were washed with PBS and incubated with the refolded protein (100 *μ*l, 50 *μ*g/ml) in maintenance medium (MEM, 1% FCS, and gentamicin) for 30 min at 37°C. Then cells were washed and incubated with DENV2 (MOI = 3, 50 *μ*l), containing the refolded protein at the same concentration, for 1 h at 37°C in maintenance medium. Cells were again washed, replenished with maintenance medium, and incubated at 37°C, 5% CO_2_. After 5 days, cytopathic effect was quantified using chemiluminescent based ATP detection (CellTiter-Glo® cell viability assay, Promega).

### 2.5. Biopanning

Phage display was performed using a Ph.D.-7 Phage Display Peptide Library according to the manufacturer's instructions. Four rounds of panning were done using two targets, the full DENV2 envelope, or the recombinant DIII obtained as described above. For each panning step, several wells in ninety-six-well plates were coated overnight at 4°C and then blocked with 400 *μ*l of 0.1 M NaHCO_3_ (pH 8.6) and 5 mg/ml BSA for 1 h at 4°C. After six washes with TBST (TBS, 0.1% Tween 20), 100 *μ*l of phages in TBST, containing 10^11^ pfu were allowed to bind for 1 h at RT. Then wells were washed ten times with TBST and phages eluted with 0.2 M glycine-HCl pH 2.2, 1 mg/ml BSA. After neutralization with Tris pH 9.1, phages were titered and bulk amplified in* E. coli* for next round. In order to increase the stringency of the selection, successive rounds were done increasing the concentration of Tween-20 during washes and reducing the amount of target fixed to the solid phase. After the fourth round, eluted phages were plated, picked, and propagated for subsequent phage-ELISA and sequencing.

### 2.6. Phage-ELISA

DENV2 E, DIII, and BSA were used to coat 96-well microtiter plate (MaxiSorp, Nunc) overnight at 4°C. Plates were blocked with TBS-BSA, washed with TBST, and incubated with 10^12^ pfu of individual phages. After washing, HRP-conjugated anti-M13 monoclonal antibody (GE Healthcare, USA) was added, incubated, and washed. Color was developed by adding TMB substrate solution for 10 min and stopped with 1 N HCl. Plates were read at 405 nm. Phage clones with the best target-to-background signal ratio were selected for DNA sequencing. The DNA insert was determined by cycle sequencing using a 96-gIII primer as recommended by manufacturer. Sequence logos and residue coloring was done as implemented in JalView v.2.10.1 [23]. Coloring was following Zappo scheme, where residues are colored according to their physicochemical properties as follows: pink, aliphatic/hydrophobic; orange, aromatic; red, positive; green, negative; blue, hydrophilic; magenta, proline/glycine; and yellow, cysteine.

### 2.7. Viral Plaque Reduction Assay

Vero cells were seeded (2 × 10^5^ cells per well) in 6-well plates and incubated overnight. The peptides were prepared at several dilutions and mixed with 100 pfu of DENV2, for a final volume of 150 *μ*l. Mixtures were incubated at RT for 1 h and then added to cell monolayers and incubated 1 h at 37°C for adsorption. Then inoculum was removed and cells were washed and overlaid with maintenance medium with 1% methylcellulose and incubated for 5 days. Then medium was aspirated; cells were fixed with 10% formaldehyde and stained with 1% crystal violet. The viral plaques were visually counted and values from experimental groups compared to virus alone, nontreated controls.

### 2.8. Peptide Cytotoxicity Assay

Peptides were incubated with cells exactly as described in the viral plaque reduction assay (1 h for 37°C) and then washed away and cells refed with maintenance medium and incubated at 37°C for 24 h. Then cytotoxicity was estimated by measuring cellular ATP using luminescence as described by the manufacturer of the CellTiter-Glo® cell viability assay. Viability was estimated by comparing readings of peptide-treated cells with those of nontreated cells.

### 2.9. Peptide-Protein Docking Simulations

The proteins structures used for the peptide-docking analysis were either obtained from the database of protein structures (in the case of the DENV envelope protein, PDB 1OAN) or obtained in our lab by homology modeling (for DIII). Sequence corresponding to the expressed protein was used to obtain the structure by homology modeling using I-TASSER web server [24]. The best model predicted by I-TASSER presented good predictive value (*C*-score = 0.77) that was structurally closely related to several viral envelope glycoproteins in the PDB database. To study the probable binding mode of the active peptides, we performed computational docking analysis using CABS-dock web server with the default parameters except for the increase of simulation cycles to 200 [25]. The best poses, ranked according to trajectory characteristics, were further analyzed to describe intermolecular interactions using LigPlot^+^ [26].

### 2.10. Statistical Analysis

Where indicated, medians were compared between the nontreated control and all other groups using Kruskal-Wallis with Dunn's multiple comparison test, as implemented in GraphPad Prism software.

## 3. Results and Discussion

### 3.1. Expression, Purification, and Refolding of Recombinant DENV2 Envelope Domain III

The dengue virus envelope is a class II virus fusion protein. This protein is a *β* strand rich, elongated molecule with three ectodomains, the centrally located DI, the apical DII which bears the fusion loop, and the Ig-like DIII, which is connected to the short, stem, and transmembrane C-terminus [14, 27]. DIII undergoes drastic repositioning during transition to the fusogenic conformation of the E protein, leading to rearrangement from dimeric to trimeric form [15, 28]. DENV E domain III forms an Ig-like *β*-barrel structure that is stabilized by one disulfide bridge.

The DIII is a key part of the envelope, as it is the receptor binding domain [13, 29]. It is also very antigenic, and antibodies to this domain are able to efficiently neutralize virus infection [18, 30]. Taking into account the important role of the envelope protein, as well as its domain III, we decided to search for peptides with binding activity to this protein, in an attempt to find novel molecules able to impair the infection process. For this purpose, we selected phage display as a robust methodology that allows the rapid screening and selection of millions of peptidic variants for binding capacity to a variety of targets. As the full envelope glycoprotein is a large protein that may present many possible nonproductive binding sites, we chose also to screen the random peptide library with domain III obtained in our lab.

**Figure 1:**
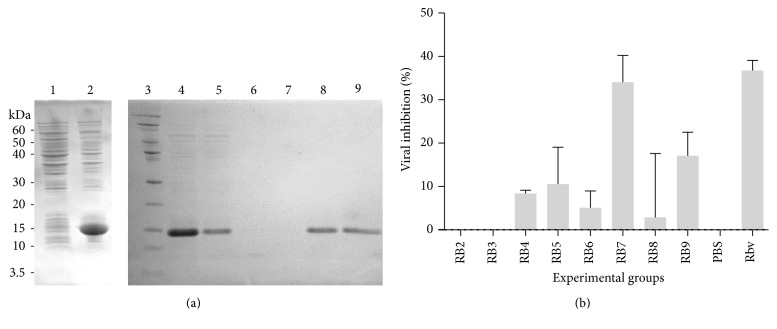
Cloning, expression in* E. coli,* and purification of codon optimized, dengue 2 envelope domain III. (a) SDS-PAGE showing several steps of protein expression and purification. 1, total lysate of cells before induction; 2, total lysate of cell after IPTG induction; 3, protein molecular weight standards; 4, solubilized inclusion bodies before loading into Ni-NTA column; 5, column flow-through; 6-7, two successive washes; 8-9, two successive elution. (b) Activity of purified and refolded recombinant domain III against DENV2 in Vero cells. Protein was refolded using several refolding conditions (RB1 to RB9) before dialysis and filtration for testing against the virus, in parallel with virus alone and ribavirin as a control. Data is presented as the mean and SD of two independent experiments. Condition RB1 was not used in this experiment as protein consistently precipitated.

The region coding for domain III of the envelope protein of DENV2 was obtained as a synthetic fragment from a commercial source after codon optimization for* E. coli* expression. This fragment was subcloned into the* E. coli* expression vector pET-30b under the control of a T7 promoter and fused to a 6xHis tail at the C-terminal. After IPTG induction,* E. coli* cultures showed high level of expression of the recombinant protein (Figure 1(a)), accounting for more than 50% of total protein, as judged from densitometric analysis of Coomassie stained SDS-PAGE gels (data not shown). As the recombinant protein was mainly present in insoluble aggregates, the purification protocol included an isolation and wash of inclusion bodies, followed by an affinity purification by affinity Ni-NTA chromatography under denaturing conditions. The affinity purification procedure implemented here allowed a high purity preparation of DIII (Figure 1(a)).

Several attempts to refold this protein by dilution into PBS failed due to precipitation; therefore the refolding conditions for this protein were further explored using several experimental conditions. These tests included varying concentrations of refolding agents and additives, such as L-arginine, reduced and oxidized glutathione, and/or polyethylene glycol [31]. It has been shown that the dengue envelope DIII may act as a dominant negative inhibitor of the infection process [10, 32–35]; therefore we decided to use this criterion to assess the functionality of the refolded DIII. After the refolding tests, each protein preparation was dialyzed against TBS, filter sterilized, and tested in a DENV2 virus binding blocking assay in Vero cells. We found that recombinant protein refolded in buffer RB7 (1.1 M guanidine, 55 mM Tris-HCl, pH 8.2, 21 mM NaCl, 0.88 mM KCl, 1 mM GSH, and 1 mM EDTA) showed a significant inhibition of the infection (Figure 1(b)) while there were no signs of cytotoxicity (data not shown), suggesting that the protein was folded and functional. This procedure was then used at a higher scale to isolate the recombinant EDIII for the biopanning and further steps.

### 3.2. Selection of Phage-Displayed Envelope Binding Peptides

**Figure 2:**
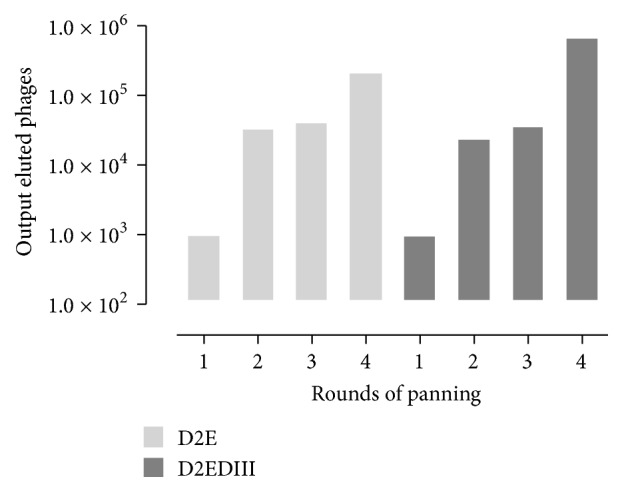
Eluted phages were enriched in successive rounds or panning. During each panning, 10^11^ input phages were incubated with the target proteins (full DENV2 envelope or domain III) and eluted as described in Materials and Methods. The eluted phages were neutralized and titer was estimated. Data is presented as the absolute number of eluted phages at each step.

In order to select for peptidic binders to both targets, we used a random library of heptapeptides presented in the M13 phage pIII protein. These phages would contain 1 to 5 copies of the peptides per capsid, theoretically allowing for the selection of higher affinity binders. As baits for the panning procedure, two sources of proteins were used, (1) a recombinant, insect-expressed DENV2 envelope and (2) recombinant,* E. coli* expressed domain III. The panning procedure involved four rounds of phage selection on solid phase-bound protein and subsequent elution and amplification. During panning progression against both proteins, the amounts of eluted phages increase stepwise (Figure 2), suggesting an effective enrichment of particular phages. After final round, 24 phages were randomly selected from each panning scheme, propagated, and purified to test their binding ability to the respective bait by phage-ELISA.

**Figure 3:**
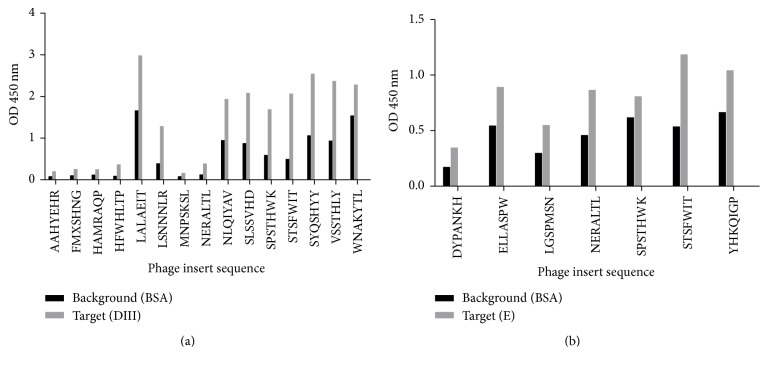
In vitro binding of selected clones as shown by phage-ELISA. Absorbance values by ELISA when phages are incubated in the presence of solid phase-bound specific ligand (target) or unspecific ligand (bovine serum albumin, as background). The binding of phages was revealed by anti-M13 antibody. (a) Results of phage-ELISA using E domain III as target. (b) Results obtained when using full E glycoprotein as target.

The analysis of eluted phages by ELISA showed that many appear to bind in a specific manner, indicated by absorbance values higher for the target as compared to those against the background (BSA) (Figure 3). Out of 24 randomly picked phages from each panning, 12 were confirmed for the full E glycoprotein and 21 were positive for the E domain III fragment. This behavior was shown by phages bearing the peptides STSFWIT, NERALTL, ELLASPW, SPSTHWK, LALAEIT, NLQIYAV, and SLSSVHD. Some other clones did not show good target-to-background signal ratio and were not used for sequencing or subsequent analyses. This result is not unexpected, as panning procedure may yield artifact binding phages which later do not bind well in the context of the ELISA.

**Figure 4:**
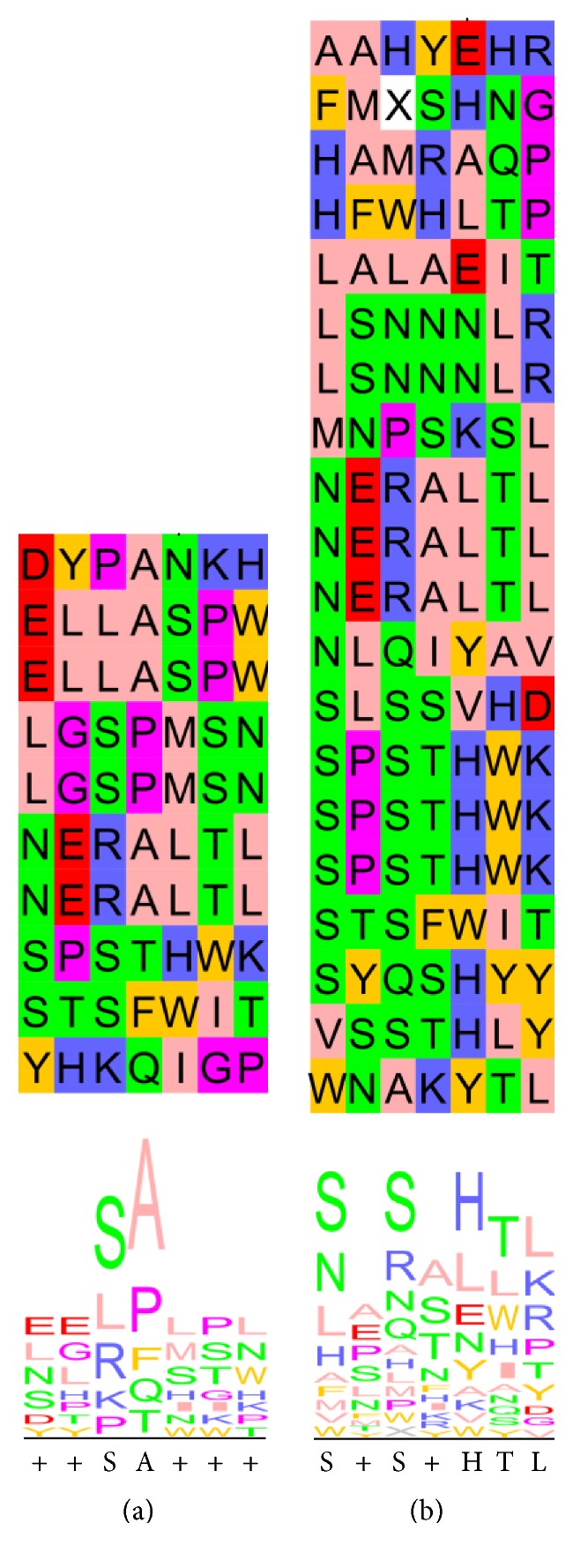
Peptide sequences resulting from binder phages confirmed by phage-ELISA. (a) Peptides resulting from panning on full DENV2 envelope. (b) Peptides resulting from panning on recombinant DENV2 envelope domain III. Sequence logos and consensus sequence, as calculated using JalView, are depicted below each list of peptides. Coloring of residues and sequence logos as per Zappo scheme, where residues are colored according to their physicochemical properties as follows: pink, aliphatic/hydrophobic; orange, aromatic; red, positive; green, negative; blue, hydrophilic; magenta, proline/glycine; and yellow, cysteine.

The phage-ELISA positive phages, 33 in total, were submitted to DNA sequencing of the randomized region. DNA sequencing revealed that binding phages carried peptides with some common features, with similar consensus sequences for the envelope glycoprotein (SxSAxxx) and for the domain III protein (SxSxHTL) (Table 1, Figure 4). The corresponding peptides had abundance of negatively charged residues, intercalated with positively charged, hydrophilic, and aliphatic/hydrophobic residues (Figure 4). The resulting peptide sequences are consistent with one of the proteins (DIII) being a smaller portion of the other (E), as the consensus sequences share some resemblance, and the consensus for peptides selected against DIII appears to be more defined. Interestingly, several phages bearing the same peptide were selected with both proteins and appeared to be repeated in the final round elution (Table 1). The fact that some phages were observed several times is also congruent with a successful enrichment of specific binders during the panning steps.

Some of the binding phages shown here carried peptide sequences already reported at the biopanning data bank, a repository that, at the moment of writing this paper, had more than 23,700 sequences appearing in biopanning data [36]. The peptides SPSTHWK and WNAKYTL were also previously reported to bind crystalline Ni3B nanoparticles [37]. Also, the peptide NERALTL appeared twice in the database, with binding activity to epoxy covered surfaces [38] and to the fusion protein of the infectious salmon anemia virus [39]. Additionally, the peptide LSNNNLR was previously reported to bind poly(dimethylsiloxane) [40].

### 3.3. Activity of Selected Peptides and Possible Mode of Binding

**Figure 5:**
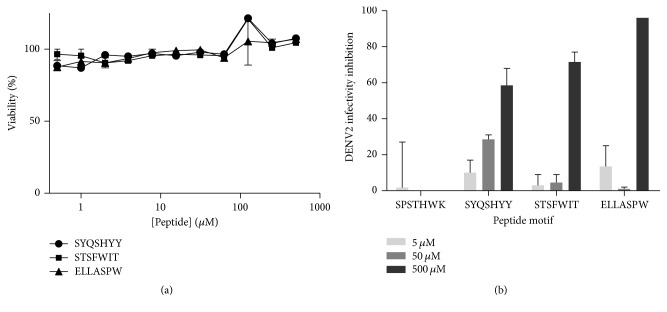
Biological activities of active peptides in Vero cells. (a) Cytotoxicity of active peptides. Cells were incubated with increasing concentrations of the peptide for 1 h and washed away and cells incubated in maintenance medium for 24. Then cytotoxicity was estimated from ATP levels using luminescence. The viability was estimated by comparing with nontreated cells. Data is presented as means and standard deviations of two independent experiments. Medians were compared between the nontreated control and all other groups using Kruskal-Wallis with Dunn's multiple comparison test. (b) DENV2 infectivity inhibition by peptides, as shown in a plaque reduction assay. The reduction in the number of plaques was estimated by comparison with virus-infected cells alone. The peptide SPSTHWK is presented as negative control since it showed no consistent inhibitory activity against the virus. Data is presented as means and standard deviations of two independent experiments.

Peptides from ELISA verified binding phages were synthesized containing the additional Gly-Gly-Gly-Ser spacer at the C-terminal and amidation of the carboxylate to block the negative charge, as suggested by the manufacturer to mimic the context of presentation in the M13 pIII protein. Then synthetic peptides were tested to check their ability to impair the infection of DENV2 in Vero cells. The synthetic peptides ELLASPW, SYQSHYY, and STSFWIT showed inhibitory activity against DENV2 infection in a plaque reduction test (Figure 5(b)). Some peptides that showed good in vitro binding to their target proteins did not show consistent inhibitory activity against the virus, that is, the peptide SPSTHWK, and were considered negative for viral inhibition (Figure 5(b)). Since the peptides were incubated only during the adsorption period (1 h at 37°C), it may be possible that the observed inhibition is due to interference in this initial step of the virus life cycle or due to virucidal activity. More experiments are required to elucidate the exact mechanism of action of these peptides. When the active peptides were incubated alone with Vero cells in the same conditions as those of the viral infection, no sign of cytotoxicity was observed (Figure 5(a)).

The fact that some other peptides with good binding activity at the panning and ELISA tests did not inhibit the DENV2 infection is not surprising, as some of these small peptides might be binding to target regions that are not critical for their functions during binding and entry. It may be also possible that some of these peptides interact differently with their targets in their synthetic form compared with the case when they were presented in the context of the phage protein pIII.

In order to explore possible modes of binding of these peptides to their targets, we performed computational docking analysis using CABS-dock online server [25]. The algorithm pipeline followed by this software allows flexible, fully blind protein-peptide docking, finishing with all atom reconstruction and optimization of the best poses [41, 42]. The structures of the proteins used for the docking were either obtained from the database of protein structures (in the case of the DENV2 envelope protein, PDB 1OAN) or obtained in our lab by homology modeling (in the case of the DENV2 EDIII).

**Figure 6:**
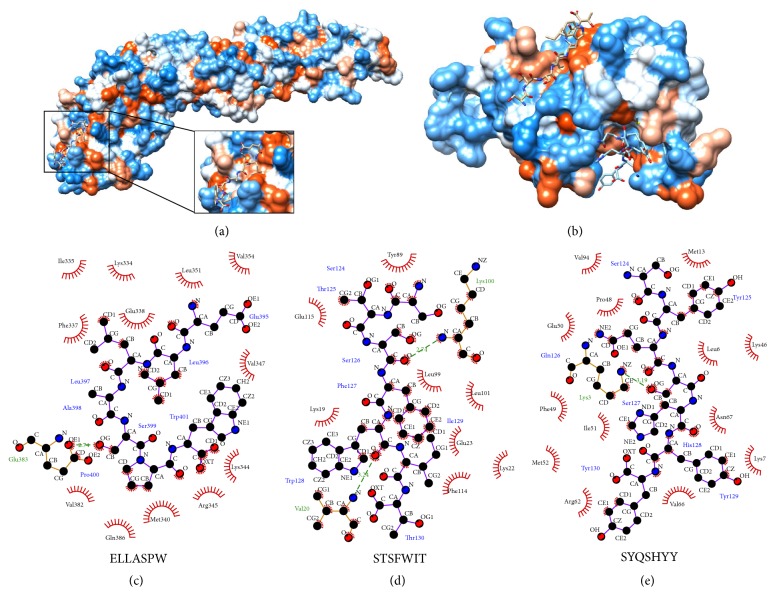
Probable binding modes of active peptides on their targets, as explored by molecular docking simulation. (a) Most favorable pose of peptide ELLASPW on the DENV2 envelope. Inset shows more details of putative binding site. (b) Most favorable poses of peptides STSFWIT and SYQSHYY on domain III. Peptide STSFWIT occupies the upper binding site of the molecule in this diagram. Proteins are depicted in their hydrophobicity surfaces, while peptides are in stick model. (c) Interaction diagram for ELLASPW-envelope. (d) Interaction diagram for STSFWIT-domain III. (e) Interaction diagram for SYQSHYY-domain III. Interaction diagrams were done in LigPlot+. Intermolecular hydrogen bonds are indicated by green dashed lines, while hydrophobic interactions are represented by arcs and radiating spokes. Pose figures were prepared using UCSF Chimera.

The docking simulations allowed prediction of possible poses for the binding of the active peptides to their target proteins. The best models predicted indicated a predominance of hydrophobic interactions over hydrogen bonds (Figure 6). Interestingly, the best pose predicted for the envelope glycoprotein and the corresponding peptide ELLASPW proposed a binding site also at the domain III of the protein (Figure 6). The suggested pose with the lowest interaction energy for the peptide ELLASPW on the envelope glycoprotein indicates hydrophobic interactions of the peptide with the protein residues I335, K334, L351, V354, F337, E338, V347, K344, R345, M340, Q386, and V382 and a hydrogen bond with E383. The suggested interactions of peptide STSFWIT with DIII include hydrophobic links with Y377, E403, L387, L389, K307, E311, K310, and F402 and hydrogen bonds with K388 and V308. Similarly, the best pose proposed for peptide SYQSHYY includes hydrophobic interactions with V382, M301, P336, E338, L294, K334, F337, I339, N355, M340, K295, V354, and R350 and hydrogen bond with K291.

Domain III of the dengue envelope has been shown to be involved in the initial steps of the binding of the virion to the receptor/attachment factors, particularly the amino acids in the sequence 380-IGVEPGQLKL-389 [13, 43, 44]. Interestingly, the best poses proposed by the simulation indicate some overlap with this region. The peptide STSFWIT appears to bind a region of DIII overlapping the 380–389 sequence, with three of its amino acids interacting with the peptide: L387, K388, and L389. The peptide ELLASPW does not appear to block or interfere with the receptor binding sequence, although two of its residues do participate in hydrophobic interactions with the peptide, E383 and Q386. The peptide SYQSHYY does not bind overlapping the receptor binding region, although one of its amino acids does interact with the peptide (V382). Interestingly, this last peptide also appears to bind very close to the sequence DKLQLK, interacting with the residues K291, Q293, and L294. This binding site may be relevant to the entry process as lysine 291 and lysine 295 were shown to be key for binding to the cellular GAGs [45]. Although best poses proposed here for the interactions between these peptides and their targets show favorable interaction energy profiles (data not shown), it should be also noted that, due to the complexities of modeling small peptides, there may be other valid binding sites. Further studies may be required to validate the proposed binding modes of the peptides and perform a rational, structure directed improvement of their biological activities.

The phage display technique has been used by several authors to search for interacting peptides, with the ultimate objective of developing new drugs against several viruses. Some examples include peptides described with activity against HIV [21, 46], Puumala virus [47], West Nile virus [48], Newcastle virus [49, 50], hepatitis C virus [51, 52], porcine reproductive and respiratory syndrome virus [53, 54], infectious bronchitis virus [55], hepatitis B virus [56], Mink enteritis virus [57], Japanese encephalitis virus [58], classical swine fever virus [59], and influenza virus [60].

Regarding peptidic inhibitors of dengue virus, three main strategies have been followed: (1) in silico designed peptides, (2) peptides mimicking the viral protein sequences, and (3) peptides obtained by panning against viral proteins. While the development of antiviral peptides against DENV has been dominated by reports using the first two approaches [61–64], a few attempts have been made to make use of the versatile phage display technology to find new antivirals. The peptides identified using random peptide libraries include those reported by Panya et al., 2014 [65], which target the envelope hydrophobic pocket, and those reported by Chew et al., 2015 [66], which target the full DENV2 virion. The peptides reported here do not share homology with the ones found by these authors, although results may not be fully comparable as different targets and libraries were used.

## 4. Conclusions

In conclusion, we have used a novel strategy to identify inhibitors of the DENV2 infectivity and we have found three peptides out of a random peptide library, which are able to specifically bind viral envelope protein and inhibit infectivity in vitro, without showing toxicity to the cells. The binding modes suggested by blind docking simulations indicate that the interactions between these active peptides and their targets may be stabilized by hydrophobic interactions, providing information relevant to the future improvement of new antivirals against this important virus.

## Figures and Tables

**Table 1:** Peptide sequences at the randomized region and frequency of appearance of bearing phages.

Target: envelope		Target: domain III	
Peptide	Frequency (%)	Peptide	Frequency (%)
DYPANKH	10	AAHYEHR	5
ELLASPW	20	FMXSHNG	5
LGSPMSN	20	HAMRAQP	5
NERALTL	20	HFWHLTP	5
SPSTHWK	10	LALAEIT	5
STSFWIT	10	LSNNNLR	10
YHKQIGP	10	MNPSKSL	5
		NERALTL	15
		NLQIYAV	5
		SLSSVHD	5
		SPSTHWK	15
		STSFWIT	5
		SYQSHYY	5
		VSSTHLY	5
		WNAKYTL	5
